# Periodontal disease in pregnancy and adverse pregnancy outcomes: Progress in related mechanisms and management strategies

**DOI:** 10.3389/fmed.2022.963956

**Published:** 2022-10-25

**Authors:** Mi Nannan, Lin Xiaoping, Jin Ying

**Affiliations:** Department of Stomatology, Shengjing Hospital of China Medical University, Shenyang, China

**Keywords:** periodontal disease, adverse pregnancy outcomes, antiphospholipid syndrome, genetic susceptibility, nutrition, periodontal therapy

## Abstract

Periodontal disease is an inflammatory and destructive disease of tissues supporting the tooth. A large number of studies have confirmed that periodontal pathogens and their metabolites can lead to adverse pregnancy outcomes in direct or indirect ways. Adverse pregnancy outcomes, such as preterm birth, low birth weight, and pre-eclampsia, have a serious impact on human reproductive health. In recent years, although the level of global medical technology has gradually improved, the incidence of adverse pregnancy outcomes has not declined and is still a global public health problem. The purpose of this review is to summarize the current data on periodontal disease in pregnancy and adverse pregnancy outcomes, including the association between periodontal disease and adverse pregnancy outcomes, the pathogenic mechanism related to this association, the efficacy of different nutrition supplements for both periodontal disease and adverse pregnancy outcomes and the effect of providing periodontal treatment on the occurrence of adverse pregnancy outcomes, to provide guidance for the prevention and treatment of adverse pregnancy outcomes in clinical practice.

## Introduction

Periodontal disease, characterized by the destruction of soft and hard tissues supporting the tooth, is a chronic infectious disease. According to data, the prevalence of pregnancy gingivitis worldwide is 30–100% (1), and among Chinese women aged 37–44, the calculus detection rate is 95.5% and the detection rate of gingival bleeding is 86.8% (2), indicating that the morbidity of periodontal disease is fairly high in women of childbearing age. Moreover, periodontal pathogens initiate chronic damage to tooth-supporting tissues, and periodontal pathogens and their metabolites can also lead to adverse pregnancy outcomes through direct or indirect pathways.

Adverse pregnancy outcomes include preterm birth, low birth weight, pre-eclampsia, fetal growth restriction, etc. Preterm birth is the main cause of perinatal morbidity and mortality, accounting for approximately 75% of perinatal mortality and more than 50% of long-term morbidity (3, 4). The number of preterm births has increased to approximately 15 million cases per year, and the incidence of preterm birth is 5–9% in Europe, 12% in USA and up to 15% in the developing world (5). Low birth weight, defined as a baby born with a weight less than 2,500 g, impacts more than 20 million babies in the world every year, and its incidence rate is approximately 14.6%, of which 91% are from low- and middle-income countries, mainly Southern Asia and sub-Saharan African countries (6). Pre-eclampsia is an idiopathic multisystem disease of pregnancy, featuring new-onset hypertension with proteinuria after 20 weeks of gestation in women and affecting 5–8% of pregnant women (7). According to the WHO, pre-eclampsia is the third cause of maternal death and is responsible for 76,000 maternal deaths each year, accounting for 16% of the global maternal mortality rate (8). Additionally, in USA, pre-eclampsia is associated with 20% of pregnancy-related maternal deaths and more than one-third of iatrogenic preterm births (7). In recent years, although the level of global medical technology has gradually improved, the incidence of adverse pregnancy outcomes has not consequently decreased and has had a serious influence on human reproductive health, which results in a huge economic burden on society and families, thus it is still a global public health problem that urgently needs to be solved today.

This review summarizes the relevant data on periodontal disease and adverse pregnancy outcomes and further explores the potential association mechanism between them to offer some assistance for the prevention and treatment of adverse pregnancy outcomes in clinical practice.

## The association between periodontal disease and adverse pregnancy outcomes

Offenbacher et al. (9) first pointed out that periodontal disease was one of the risk factors for premature low birth weight through a case-control study of 124 pregnant women since 1996, which has initiated a series of relevant studies to explore the association between periodontal disease and adverse pregnancy outcomes. A review analyzed 232 relevant clinical research studies from 1996 to April 2020 and found that most of the studies demonstrated that periodontal disease had an evident association with multiple adverse pregnancy outcomes (10).

It is noteworthy that most of the research, in the last 2 years, also supported a significant association between periodontal disease and adverse pregnancy outcomes. Several studies suggested that periodontal disease during pregnancy was related to mothers giving birth to premature babies, and this correlation became more obvious with the aggravation of maternal periodontal inflammation (11–13). Both a meta-analysis and a cohort study showed that, compared with pregnant women without periodontitis, pregnant women with periodontitis had a doubled risk of preterm birth (14, 15). Similarly, a retrospective case-control study enrolling 555 postpartum women found that pregnant women with periodontitis were six times more likely to deliver premature babies than women without periodontitis (16). Likewise, a hospital-based cross-sectional case-control study suggested that the presence of postpartum maternal periodontitis and its severity remained independent risk factors for preterm birth in the presence of antepartum smoking habits and routes of delivery (17). A cross-sectional study recording clinical periodontal parameters and assessing serum C-reactive protein levels confirmed that poor maternal periodontal status, increased oral inflammatory burden and increased systemic inflammation had an adverse effect on infant birth weight (18). In addition, it was suggested that periodontal infection could exacerbate the progression of pre-eclampsia, and periodontal disease was considered to be one of the risk factors for pre-eclampsia (19, 20).

Otherwise, there are a small number of studies that do not support a significant correlation between periodontal disease and adverse pregnancy outcomes. A case-control study, performed by collecting subgingival biofilm samples of pregnant women from four sites up to 48 h postpartum and processing by polymerase chain reaction for the presence of periodontal pathogens, showed that most periodontal pathogens in the oral cavity were not apparently connected with the development of preterm birth or low birth weight (21). A cohort study that was made on 159 Caucasian Spanish pregnant women also indicated no statistical association between periodontal disease and preterm birth (22).

At present, the association between periodontal disease and adverse pregnancy outcomes remains controversial, may be due to the following factors: the different sample sizes, races, ages, living habits and socioeconomic statuses, the different definitions of periodontal disease and adverse pregnancy outcomes in the studies.

Therefore, future studies should expand the sample size and exclude the influence of other factors on adverse pregnancy outcomes, and more uniform and clear diagnostic criteria for periodontal disease with adverse pregnancy outcomes should be established.

## Mechanisms related to periodontal disease leading to adverse pregnancy outcomes

It is generally accepted that periodontal pathogens and their metabolites can affect progressive and inflammatory lesions of tooth-supporting tissues by regulating the release of inflammatory mediators, and periodontal pathogens and inflammatory mediators are capable of spreading to the placenta and contributing to various adverse pregnancy outcomes (3). However, the underlying mechanism is not yet clear, scholars have attempted to explain the mechanisms of periodontal disease causing adverse pregnancy outcomes from the following aspects.

### The influence of periodontal pathogen infection

As the pathogenesis of adverse pregnancy outcomes, intrauterine infection can lead to 25–40% of preterm birth incidence, which is closely related to periodontal pathogens (4). Periodontal pathogens invade the uterus *via* hematogenous dissemination, causing adverse pregnancy outcomes. Currently, the periodontal pathogens found in the fetal-placenta unit include *Aggregatibacter actinomycetemcomitans*, *Prevotella intermedia*, *Campylobacter rectus*, *Tannerella forsythia*, *Treponema denticola*, *Fusobacterium nucleatum*, and *Porphyromonas gingivalis*, and the most common species are *P. gingivalis* and *F. nucleatum* (23, 24).

#### *Porphyromonas gingivalis* and adverse pregnancy outcomes

*Porphyromonas gingivalis* is one of the most important periodontal pathogens in the oral cavity and is associated with many adverse pregnancy outcomes. On the one hand, its unique virulence factors, surface adhesions and enzymes elicit direct or indirect damage in different ways to fetal and maternal tissue, resulting in dysfunction of the maternal endothelium and eventually leading to the occurrence of systemic inflammatory responses (25). In addition, it has been proposed that *P. gingivalis* could be detected in the placental villus stroma of premature women and in the umbilical cord of premature infants, and *P. gingivalis* in the umbilical cord was closely related to pre-eclampsia (26). On the other hand, *P. gingivalis*, by activating the JNK and P38 signaling pathways and inhibiting the PI3K/Akt signaling pathway, stimulates apoptosis and the inhibition of invasiveness of extrachorionic trophoblast cells and results in insufficient remodeling of the uterine spiral artery, which can lead to fetal insufficiency of blood supply, finally causing fetal malnutrition and even death (27, 28).

In maternal-fetal immunity research, *P. gingivalis* has the ability to regulate the imbalances between different immune cells, which allows *P. gingivalis* to reinforce its persistence and survival time in maternal and fetal tissues, it also improves the ability of *P. gingivalis* to evade immune responses. *P. gingivalis* can also cause increased production of proinflammatory cytokines and activation of acute-phase responses, resulting in a shift in the maternal-fetal immune response and the development of adverse pregnancy outcomes (29, 30). In addition, it is noted that *P. gingivalis* also increases oxidative stress (OS) by activating phagocytosis mediated by neutrophils and macrophages and releasing a large amount of reactive oxygen species in the systemic circulation. In turn, the increased OS induces hypoxia and initiates the apoptotic process in maternal-fetal tissue (25). Although some degree of hypoxia and OS are required for the normal labor process, excessive hypoxia of extrachorionic trophoblastic cells, uterine epithelial cells, and endothelial cells can lead to uterine inflammation, malformed remodeling of uterine spiral arteries, and increased risk of endothelial cell damage (31).

#### *Fusobacterium nucleatum* and adverse pregnancy outcomes

*Fusobacterium nucleatum*, isolated from the vaginal microbiome, is a gram-negative anaerobic bacterium and is one of the periodontal pathogens. *F. nucleatum* is recognized to colonize the placenta with the help of outer membrane proteins such as Fusobacterium Adhesion A (FadA), fibroblast activation protein 2 (Fap2), and radiation genes (RadD) (32). Among them, Fad A helps *F. nucleatum* to weaken the binding ability between endothelial cells and invades the placenta; thus, it can easily cross the vascular endothelial cell gap and enter the blood circulation, finally diffusing into the amniotic fluid, placenta and fetus and playing an invasive pathogenic role in the mother and fetus (33). At the same time, Fap2 and RadD can enhance the interspecific co-aggregation and cell adhesion of *F. nucleatum*, while the Fap2 mutant significantly attenuates the ability of *F. nucleatum* to colonize the mouse placenta. The RadD mutant has the opposite effect, and can reinforce the ability of *F. nucleatum* to colonize the mouse placenta and reduce the fetal survival rate (34, 35). In addition, *F. nucleatum* induces placental inflammation by activating Toll-like receptors (TLRs), leading to adverse pregnancy outcomes. *In vitro* studies have shown that *F. nucleatum* activates both TLR2 and TLR4, but it induces the inflammatory response in the mouse placenta merely by activating TLR4, accompanied by neutrophil infiltration into the decidua, whereas TLR2 plays an insignificant role in the incidence of fetal loss or the inflammatory response (36).

Therefore, *P. gingivalis* and *F. nucleatum*, in the development of periodontal disease causing adverse pregnancy outcomes, play an important role in different ways. It is hoped that more studies will further explore the mechanism between other periodontal pathogens and adverse pregnancy outcomes to provide more information to reduce the number and function of periodontal pathogens, thereby lowering the incidence of adverse pregnancy outcomes.

### The influence of changes in the levels of inflammatory mediators

During the development of periodontitis, changes in the levels of different inflammatory mediators play an important role, and the levels of inflammatory mediators in saliva are positively correlated with the severity of periodontal disease. The increased levels of different inflammatory mediators, including tumor necrosis factor alpha (TNF-α), interleukin-1β (IL-1β), and interleukin-6 (IL-6), in maternal serum, myometrium, amniotic fluid and fetal membranes are one of the characteristics of normal physiological pregnancy, which will cause prostaglandin 2 (PGE2) to rise to a certain level and lead to the initiation of delivery. Advanced elevation of inflammatory mediators in the fetal-placental unit causes premature rupture of membranes and uterine contractions, which will result in premature birth or spontaneous abortion (37).

Thus, it is speculated that inflammatory mediators may work as a bridge between periodontal disease and adverse pregnancy outcomes. Regarding the pathological mechanism of inflammatory mediators between them, the consensus of the European Periodontal Union and the American Periodontal Society has pointed out that the inflammatory mediators produced by the periodontal local tissue could directly affect the fetal-placental system or reach the liver through blood circulation and increase the systemic inflammation state through acute phase protein responses, such as C−reactive protein, exerting an impact on the fetal-placental unit (38).

To explore the relevant mechanism of the association between periodontal disease and adverse pregnancy outcomes and the predictors of adverse pregnancy outcomes, most studies have examined the levels of inflammatory mediators in different body fluids, such as gingival crevicular fluid and serum in pregnant women. A case-control study of pregnant women with periodontal disease showed that, compared with women who received periodontal therapy postpartum, pregnant women receiving periodontal system therapy during pregnancy had significantly decreased levels of IL-1β, IL-6, IL-10, and IL-12p70 in gingival crevicular fluid (39). A cross-sectional study showed that the levels of IL-1β, IL-6, and PGE2 in the gingival crevicular fluid of preterm women were significantly higher than those of women with full-term pregnancy (40). Similarly, a study showed that higher levels of IL-2, IL-6, IL-10, TNF-α, and PGE2 were detected in the serum of preterm women with periodontal disease, while the serum levels of IL-2, IL-6, and IL-10 were higher in high-risk preterm women than in low-risk preterm women (37). In addition, in animal studies, it was also shown that the serum levels of IFN-γ, IL-1β, IL-6, and IL-8 in premature mice with oral infection were significantly increased (41, 42). Equally, serum C-reactive protein concentrations were significantly increased to pregnant women with periodontal disease and were positively associated with the occurrence of low birth weight and pre-eclampsia (18, 43). In summary, all of the above studies showed that, periodontal disease can increase the levels of inflammatory mediators both in the oral cavity and throughout the body, causing the systemic inflammatory response during pregnancy, and eventually leading to preterm birth, low birth weight and pre-eclampsia.

Therefore, inflammatory mediators have a certain role in the development of adverse pregnancy outcomes caused by periodontal disease, but further research is required to propose clear and specific inflammatory markers that can be used as predictors of adverse pregnancy outcomes to achieve the goal of early intervention and ultimately reduce the incidence of adverse pregnancy outcomes.

### The influence of host immune response

The imbalance between host defense capabilities and microbes and their virulence factors can aggravate the progression of diseases, which in turn initiates the host immune response. Similarly, when periodontal pathogens enter the maternal placenta, they can activate the maternal adaptive immune response and prompt mother to produce bacterial-specific antibodies, bacterial-IgM antibodies are formed firstly, and with the stimulation of inflammatory cytokines, IgM antibodies are converted into immunoglobulin G (IgG) antibodies.

Elevated IgM antibodies in fetal umbilical cord blood may be a risk factor for adverse pregnancy outcomes. Concentrations of IgM antibodies against red and orange complex bacteria were significantly increased in cord blood of preterm infants compared with term infants (44). Meanwhile, anti-*C. rectus* IgM titers in fetal umbilical cord blood were the best predictor of adverse pregnancy outcomes (38). Compared with women who received periodontal non-surgical treatment during pregnancy, the fetuses, delivered by the mothers in the control group, presented the significantly higher risk of adverse pregnancy outcomes and higher levels of anti-periodontal pathogenic bacteria IgM antibodies in the umbilical cord blood (45).

Immunoglobulin G is the main bacterial antibody product when tooth-supporting tissues are infected, but the effect of elevated IgG antibodies against periodontal pathogens in maternal serum on adverse pregnancy outcomes is still controversial. Some scholars have pointed out that IgG antibodies against periodontal pathogens had a protective effect on adverse pregnancy outcomes, lower serum concentrations of anti-*A. actinomycetemcomitans*, *F. nucleatum*, and *P. gingivalis* IgG antibodies increased the risk of preterm birth and low birth weight (46, 47). Women had periodontal disease with low titers of anti-*P. gingivalis* IgG in serum demonstrated a 7-fold higher risk of preterm birth than those with high titers (48). Nevertheless, others have also shown that IgG antibodies against periodontal pathogens might be a risk factor for adverse pregnancy outcomes. Compared with term mothers, higher levels of anti-*P. gingivalis* antibodies could be detected in the serum of mothers who delivered premature low birth weight infants (49, 50). Furthermore, FcγRIIB gene polymorphism may be related to this debate, FcγRIIB is the sole inhibitory receptor in IgG-Fc receptors and is able to inhibit periodontal bacteria by expressing serum IgG and peripheral blood bone marrow-dependent lymphocytes. In people with chronic periodontitis, carriers of the FcγRIIB-nt645 + 25AA genotype showed a remarkably higher level of clinical attachment loss than carriers of the FcγRIIIB-nt645 + 25GG genotype and a significantly lower IgG response to *P. gingivalis* (51). Studies suggested that the FcγRIIB-nt645 + 25A/G gene polymorphism was also associated with pre-eclampsia and gestational hypertension, and compared to the FcγRIIB-nt645 + 25AG and/or GG genotype or the G allele, the FcγRIIB-nt645 + 25A genotype or A allele demonstrated a higher frequency in women with pre-eclampsia (52).

In brief, different host immune responses to periodontal pathogens have diverse effects on the incidence of adverse pregnancy outcomes. Future studies should verify the specific role of host immune response in this process.

### The influence of antiphospholipid syndrome with elevated anticardiolipin antibodies

Antiphospholipid syndrome (APS) that is mediated by antiphospholipid antibodies can lead to adverse pregnancy outcomes. Anticardiolipin antibodies (aCLs) are one of the characteristic antibodies of APS and can also be found in the serum of 15–20% of patients with periodontitis at concentrations exceeding those presented in 95% of the healthy population (53). It has been proposed that periodontal disease with elevated aCL levels could result in adverse pregnancy outcomes through the following pathways.

It is well known that the target antigen of aCL is protein β2 glycoprotein-I (β2GPI), which has a positive effect on inhibiting blood coagulation in APS patients. The combination of aCL and β2GPI can not only promote the activation of complement, endothelial cells, inflammatory cells, and platelets but can also interfere with the normal function of trophoblast cells and decidual cells, contributing to thrombosis and adverse pregnancy outcomes (54). By searching the Swiss-Prot database, it was found that the periodontal pathogens *P. gingivalis*, *T. denticola*, and *A. actinomycetemcomitans* had peptide chains that are highly similar to TLRVYK, the marker peptide chain of β2GPI; thus, there was a possibility of molecular mimicry between periodontal pathogens and β2GPI (55). Animal experiments have confirmed that after inoculation with *P. gingivalis*, which has a peptide chain structure similar to that of TLRVYK, the antibodies produced in mice cross-react with β2GPI to form a β2GPI complex, which could give rise to thrombosis, abortion and premature birth (56).

It was noted that aCL could also influence adverse pregnancy outcomes by causing competition between annexin V and periodontal pathogens. Annexin V is a phospholipid-binding protein that binds to the cell membrane with exposed anionic phospholipids and forms a protective barrier on the surface of cells such as trophoblast cells and endothelial cells, exerting the ability to inhibit fibrin clot formation and promote cell membrane repair and resealing. However, in patients with periodontal disease, periodontal pathogens can compete with annexin V for the binding site of aCL in serum because they have a structure similar to the marker peptide chain of β2GPI, thus destroying the protective function of annexin V on trophoblast cells and endothelial cells and increasing the risk of adverse pregnancy outcomes (57).

Additionally, IgG aCL was regarded as having a relationship with increased serum markers of vascular inflammation. IgG was purified from the serum of periodontal disease patients with elevated aCL levels and used to stimulate endothelial cells and trophoblast cells. On the one hand, IgG aCL contributes to the inflammatory response by activating TLR4. When aCL was removed by immunoabsorption, the ability of serum IgG to activate TLR4 was significantly reduced (53). There is some disagreement regarding the interaction between aCL and TLR4, and it was suggested that aCL could improve the binding ability of β2GPI with cell surface TLR4 through a cross-linking reaction with β2GPI (58). However, *P. gingivalis* was also hypothesized to have homology to the peptide sequences of both β 2GP1 and TLR4, so aCL in the serum of patients with periodontal disease could directly bind and activate TLR4 (59). On the other hand, IgG aCL could lead to increased production of the inflammatory cytokine IL-8, and the production of IL-8 demonstrated a significant reduction when aCL was removed by immunoabsorption or when early trophoblast cells were blocked with anti-TLR4 antibody (53).

However, there are still few studies on the adverse pregnancy outcomes affected by gestational periodontal disease associated with APS; thus, future studies are needed to further explore the specific role of APS in the course and provide more explicit ideas for its prevention and treatment.

## The effect of nutritional intake on periodontal disease and adverse pregnancy outcomes during pregnancy

Exploration of related mechanisms provides potential directions for inhibiting bacterial colonization and inflammatory responses in the fetal-placental unit. Balanced nutritional intake plays an indispensable role in heightening the immunity of pregnant women and promoting safer pregnancy and childbirth; however, the world is facing the dual challenge of malnutrition, including over- and undernutrition, which has a huge impact on human health (60). Consumption of foods in high-sugar, low-fiber, high-saturated fat, and low-polyunsaturated fat diets increases the tendency of periodontal disease (61). It was found that compared to normal-weight gestational periodontitis patients, pregnant women with both periodontitis and obesity were more likely to deliver preterm infants with pre-eclampsia (62). Due to changes in the body’s metabolic function, pregnant women have increased requirements for some nutrients, so a better understanding of the effect of diet on periodontal disease and adverse pregnancy outcomes, especially a reasonable intake of a variety of nutrition is needed.

### The effect of macronutrients on periodontal disease and adverse pregnancy outcomes

Macronutrients include carbohydrates, proteins and fats. The more well-documented macronutrients associated with periodontal disease and adverse pregnancy outcomes are carbohydrates and omega-3 fatty acids.

Carbohydrates consist of sugars, starches and fibers, which have different effects on the body. Excessive intake of sugary foods is positively associated with systemic microbial dysbiosis and increased levels of inflammatory mediators, thus leading to different inflammatory responses in the body. In addition, glucose also promotes the apoptosis of periodontal ligament cells and inhibits their proliferation (63). Nevertheless, fiber foods are rich in antioxidants and other health-promoting compounds that can reduce OS and inflammatory responses in the body. In addition, they can slow the body’s absorption of carbohydrates, thereby lowering and controlling blood sugar levels. A prospective cohort study of Chinese women showed that women who consumed fewer vegetables during pregnancy had a higher risk of preterm birth (64). Similarly, it has been shown that a high-sugar diet during pregnancy was connected with the occurrence of pre-eclampsia, while daily dietary fiber intake could reduce the incidence of pre-eclampsia (65).

Omega-3 fatty acids are a class of essential polyunsaturated fatty acids that regulate the body’s inflammatory response and immune function. On the one hand, omega-3 fatty acids can reduce the level of clinical attachment loss, probing depth, and the number of inflammatory mediators in the periodontal tissue of patients with periodontitis, promoting the healing of periodontal wounds (66, 67), and they also have the ability to inhibit osteoclast cell differentiation and alveolar bone resorption induced by periodontal diseases (68). On the other hand, pregnant women with lower levels of omega-3 fatty acids tend to have a higher possibility of preterm birth and pre-eclampsia. In addition, moderate supplementation with omega-3 fatty acids reduces this risk, but excessive supplementation with omega-3 fatty acids also increases this risk (69, 70). In animal studies, omega-3 fatty acids inhibited inflammatory responses mediated by TLR2 and TLR4 in endothelial cells and reduced *F. nucleatum*-induced adverse pregnancy outcomes in mice, while supplementation with omega-3 fatty acids derived from fish oil for pregnant mice inhibited *F. nucleatum* proliferation and placental inflammation, which increased fetal and neonatal survival (71).

### The effect of micronutrients on periodontal disease and adverse pregnancy outcomes

Micronutrients are composed of vitamins, minerals, and trace elements. It is demonstrated that the vitamins A–E and the minerals calcium and zinc are related to both periodontal disease and adverse pregnancy outcomes.

Vitamin A is considered to influence the body’s gene transcription, bone metabolism, immune function, and antioxidant activity. Vitamin A deficiency is found to increase the significant risk of periodontal disease; in contrast, a higher intake of beta-carotene is connected with a decrease in the number of sites with a periodontal probing depth >3 mm (72, 73). Additionally, some additional vitamin A is required during pregnancy to assist the mother’s metabolism and support fetal growth and development (74). Vitamin A is thought to be a risk factor for pre-eclampsia, and vitamin A deficiency increases the incidence of adverse pregnancy outcomes (75).

Vitamin B complexes, including vitamins B1, B2, B3, B6, B9, and B12, have the ability to adjust the production and release of intracellular energy, and they also impact the metabolism of proteins, fats, and carbohydrates (61). On the one hand, vitamin B complex deficiency leads to a clearly lower resistance to bacterial infection. In addition, a prospective cohort study showed that increased serum vitamin B12 was negatively associated with periodontal probing depth, clinical attachment loss and the number of missing teeth in patients with periodontal disease (76). On the other hand, the lower vitamin B complex levels can affect cell growth and neural tissue development; thus, most prenatal supplements are recommended to include vitamin B complexes due to their high energy requirements. Some studies indicated that a low content of vitamins B2, B3, and B9 would enhance the probability of low birth weight and pre-eclampsia (77). Similarly, vitamin B12 deficiency was associated with an increased risk of placental rupture, stillbirth, preterm birth, and low birth weight, which affected 25% of pregnant women worldwide (78).

Vitamin C, acting as enzymatic cofactor in a range of essential metabolic reactions that include hydroxylation of proline and lysine needed to stabilize collagen structure during its manufacture, exerts a vital role in maintaining the integrity of connective tissues such as the periodontium (79). Vitamin C is also involved in inducing the differentiation of periodontal ligament stem cells, which plays a crucial role in preventing and slowing the progression of periodontal disease (80). Furthermore, Vitamins C and E have a synergistic effect to improve antioxidant defenses and inhibit free radical formation, thus exerting an essential role in OS inhibition (81). A case-control study showed that pregnant women with both periodontal disease and pre-eclampsia had significantly lower levels of vitamin C in saliva and serum (82). Similarly, vitamin E levels are inversely correlated with the severity of pre-eclampsia, and vitamin E deficiency is regarded as one of the risk factors for pre-eclampsia (75). Therefore, the combined supplementation of vitamins C and E will suppress inflammatory reactions and promote wound healing to improve periodontal status, reducing the risk of adverse pregnancy outcomes accordingly.

Vitamin D regulates calcium and phosphorus metabolism and the immune inflammatory response. A cross-sectional study found that vitamin D and calcium levels were negatively related to random blood glucose, glycated hemoglobin, probing depth, and clinical attachment loss, indicating that lower levels of vitamin D and calcium had aggravated the severity of periodontal disease (83). At the same time, the combined supplementation of vitamin D and calcium significantly reduced periodontal inflammation and C-reactive protein levels compared with single supplementation of vitamin D or calcium (84). It was also shown that compared with the control group, vitamin D and calcium supplementation significantly improved the effect of periodontal non-surgical treatment (85). In addition, the mean serum 25-hydroxyvitamin D levels in pregnant women with periodontal diseases were significantly lower than those in pregnant women with healthy periodontal tissues. Meanwhile, maternal vitamin D deficiency promotes the presence of preterm birth, low birth weight, gestational diabetes, and pre-eclampsia. It is conceivable that adequate vitamin D and calcium supplementation can not only reduce the severity of periodontal disease during pregnancy but also decrease the incidence of adverse pregnancy outcomes (86, 87).

Zinc, which inhibits the activation and expression of inflammatory cells and neutralizes bacterial toxins, is an essential trace mineral for humans. Therefore, zinc deficiency can elicit and aggravate the development of periodontal diseases (88). In addition, a lower degree of zinc is responsible for approximately 500,000 maternal and child deaths each year, and zinc deficiency during pregnancy has a positive relation to preterm birth, intrauterine growth retardation, low birth weight, prolonged delivery, and gestational hypertension (89).

### The effect of probiotics on periodontal disease and adverse pregnancy outcomes

Probiotics are exogenous active microorganisms that can effectively regulate the balance of the organism flora and control the reproduction of harmful flora. In tissues supporting the tooth, probiotics are capable of inhibiting the growth of periodontal pathogens and maintaining the periodontal microecological balance, at the same time, probiotics play a vital part in regulating the host’s immune response to periodontal pathogen infection (90, 91). In addition, probiotics that enter the gastrointestinal tract are also involved in the regulation of the host’s systemic immune function. For example, bacterial vaginosis is a common form of vaginal dysbiosis that plays an important role in the development of spontaneous preterm birth, miscarriage and endometritis, and the supplementation of probiotics improves the treatment of bacterial vaginosis by repairing the vaginal microbiota environment, thereby reducing the occurrence of premature birth and miscarriage (92, 93).

Given that both overnutrition and undernutrition supplementation during pregnancy will adversely affect the growth and development of pregnant women and fetuses, besides, nutrient supplementation has the advantages of fewer visits, extremely small side effects, non-invasive treatment, and lower cost, we advocate appropriate supplementation of different nutrition during pregnancy, which not only lessens the course of periodontal disease during pregnancy and adverse pregnancy outcomes, but also serves as an adjunct to non-surgical periodontal treatment during pregnancy (Table 1).

**TABLE 1:** The studies of the effect of nutritional intake on periodontal disease and adverse pregnancy outcomes during pregnancy.

Nutrient	References	Findings
**Macronutrients**		
Carbohydrates	Liu et al. (63)	Glucose had the effect of promoting the apoptosis of periodontal ligament cells and inhibiting their proliferation
	Lu et al. (64)	Women who consumed less vegetables during pregnancy had a higher risk of preterm birth
	Sanjarimoghaddam et al. (65)	A high-sugar diet during pregnancy was connected with the occurrence of pre-eclampsia, while daily dietary fiber intake could reduce the incidence of pre-eclampsia
Omega-3 fatty acids	Azuma et al. (66)	Omega-3 fatty acids decreased the number of inflammatory mediators in the periodontal tissue of patients with gingivitis and periodontitis
	Chatterjee et al. (67)	Omega-3 fatty acids reduced the level of clinical attachment loss, probing depth, promoting the healing of periodontal wounds
	Ozaki et al. (68)	Omega-3 fatty acids had the ability to inhibit osteoclasts cell differentiation and alveolar bone resorption induced by periodontal diseases
	Simmonds et al. (69)	Women with lower levels of omega-3 fatty acids in pregnancy tend to have a higher risk of preterm birth, moderate supplementation of omega-3 fatty acids reduced this risk
	Bakouei et al. (70)	Pregnant women with higher total omega-3 fatty acids had an decreased possibility of pre-eclampsia
	Garcia et al. (71)	Omega-3 fatty acids inhibited inflammatory responses mediated by TLR2 and TLR4 in endothelial cells and reduced *F. nucleatum*–induced adverse pregnancy outcomes in mice
**Micronutrients**		
Vitamin A	Dodington et al. (72)	The higher intake of beta-carotene was connected with a decrease in the number of sites with a periodontal probing depth >3 mm
	Duan et al. (75)	Vitamin A was thought to be a risk factor for pre-eclampsia, and vitamin A deficiency increased the incidence of adverse pregnancy outcomes
Vitamin B	Zong et al. (76)	The increased serum vitamin B12 was negatively associated with periodontal probing depth, clinical attachment and the number of missing teeth in patients with periodontal disease
	Bulloch et al. (77)	The low content of vitamins B2, B3 and B9 would enhance the probability of low birth weight and pre-eclampsia
	Rogne et al. (78)	Vitamin B12 deficiency was related to the increased risk of placental rupture, stillbirth, preterm birth, and low birth weight
Vitamin C	Varela et al. (79)	Vitamin C exerted a vital role in maintaining the integrity of connective tissues such as the periodontium
	Tada et al. (81)	Vitamin C played a role in slowering the progression of periodontal disease
	Shetty et al. (82)	Vitamin C presented the lower levels in saliva and serum in pregnant women with both periodontal disease and pre-eclampsia
Vitamin D	Agrawal et al. (83)	Vitamin D and calcium deficiency had aggravated the severity of periodontal disease
	Ferrillo et al. (86)	The lower levels vitamin D and poor oral health promoted the presence of preterm birth and low birth weight
	Taneja et al. (87)	Vitamin D deficiency increased the prevalence of pre-eclampsia, gestational diabetes mellitus and preterm birth
Vitamin E	Duan et al. (75)	Vitamin E deficiency was correlated with a higher incidence of pre-eclampsia
Calcium	Rodrigues et al. (84)	The combined supplementation of calcium and vitamin D significantly reduced periodontal inflammation and C -reactive protein levels compared with single supplementation of vitamin D or calcium
	Rodrigues et al. (85)	Calcium and vitamin D supplementation significantly improved the effect of periodontal non-surgical treatment
Zinc	Marshall et al. (89)	Zinc deficiency during pregnancy had a positive relation to preterm birth, intrauterine growth retardation, low birth weight, prolonged delivery, and gestational hypertension
Probiotics	Bustamante et al. (91)	Probiotics were capable of inhibiting the growth of periodontal pathogens and maintaining the periodontal microecological balance
	López et al. (92)	The supplementation of probiotics improved the treatment of bacterial vaginosis by repairing the vaginal microbiota environment, thereby reducing the occurrence of premature birth and miscarriage

## The effect of periodontal therapy on adverse pregnancy outcomes

Periodontal disease is considered to be one of the risk factors for adverse pregnancy outcomes. Whether periodontal treatment during pregnancy can reduce the prevalence of adverse pregnancy outcomes is still a common concern of stomatologists and obstetricians. A meta-analysis found that periodontal treatment during pregnancy decreased the incidence of preterm birth (RR = 0.78, 95% IC is 0.62–0.98, *P* = 0.03) and could significantly improve the birth weight of infants (94). Similarly, A meta-analysis evaluated 19 randomized controlled trials from 2000 to 2018 to explore the effect of periodontal treatment on adverse pregnancy outcomes, among which, 12 studies demonstrated a positive effect, while another seven studies showed no significant effect. It was found that the incidence of preterm birth ranged from 0 to 53.5% in the treatment group, while in the control group, its range was 6.38–72%, the rate of low birth weight varied from 0 to 36% in the treatment group, while it was 1.15–53.9% in the control group, thus indicating that periodontal non-surgical treatment during pregnancy was safe (95). Although it did not completely avert the rate of adverse pregnancy outcomes, it can be recommended as a part of antenatal care. Moreover, it was shown that the odds ratios for both preterm and low birth weight were 3.86 times and 2.96, respectively (96). Therefore, periodontal disease could be deemed to be one of the risk factors for adverse pregnancy outcomes, and the elimination of periodontal infection lessened the adverse pregnancy outcomes. However, there is a case-control study showing a different result, in the periodontal treatment group, a statistically apparent reduction was observed in all clinical and microbiological parameters, but there were no evident differences in the incidence of preterm birth and low birth weight, it was indicated that in Spanish Caucasian pregnant women with periodontitis, periodontal non-surgical treatment did not significantly reduce the odds of adverse pregnancy outcomes (97).

The diverse intervention methods used in these studies may be one of the reasons for the difference. In the above studies suggesting a positive effect, the intervention types mainly included: scaling and root planning with chlorhexidine; scaling and root planning with plaque control; scaling and root planning, and oral hygiene instruction with chlorhexidine; scaling and root planning with maintenance every 3 weeks until delivery. Nevertheless, among the studies showing no significant effect, the majority of interventions were scaling and root planning only, scaling and root planning and oral hygiene instruction. It is suggested that a single intervention type may be insufficient to address periodontal inflammation or control oral bacterial load, and performing a combination of multiple approaches is more effective. At the same time, maintenance of health oral status throughout the gestation is recommended, rather than the “one-time deal” (98, 99).

**FIGURE 1:**
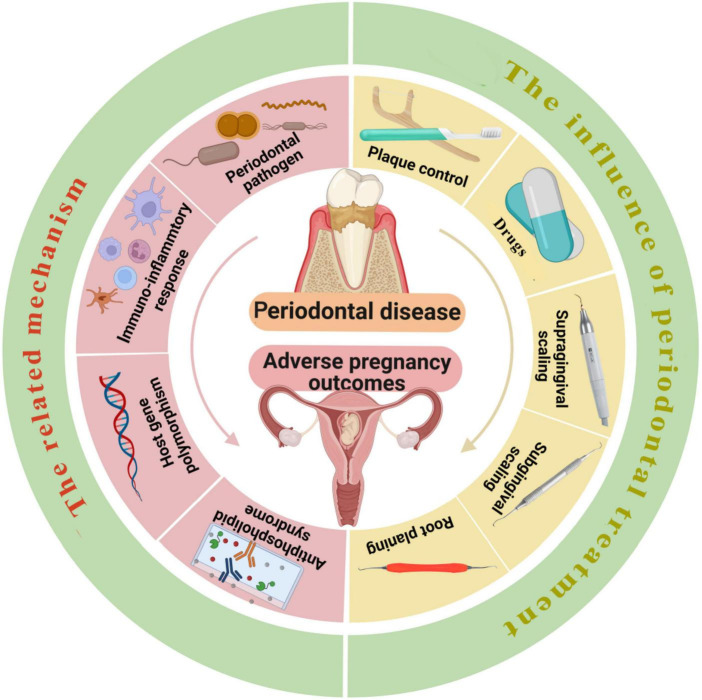
The left side of the picture shows the mechanisms related to periodontal disease leading to adverse pregnancy outcomes, including periodontal pathogen infection, the changes in the level of inflammatory mediators, host immune response, gene polymorphism, and APS with elevated anticardiolipin antibodies. The right side of the picture shows the effect of periodontal treatment on adverse pregnancy outcomes, the intervention methods include plaque control, application of drugs, supragingival scaling, subgingival scaling and root planning. Reasonable intervention of periodontal diseases during pregnancy is beneficial to the physical and mental health of patients and can reduce the mother-to-child transmission of periodontal pathogens. The ideal time for periodontal treatment is the second trimester (14–27 weeks), if intervention for infection is absolutely necessary, emergency treatment can be performed throughout pregnancy. Created with https://biorender.com/.

Currently, it has been universally acknowledged that non-surgical periodontal treatment during pregnancy is safe, although oral anti-inflammatory treatment during pregnancy did not significantly lessen adverse pregnancy outcomes (100). Furthermore, as a vital part of pregnancy health, reasonable intervention of oral diseases during pregnancy is beneficial to the physical and mental health of patients and can reduce the mother-to-child transmission of oral pathogens. Therefore, it is recommended to choose the right time under the premise of following the principles of safety, necessity, comfort and multidisciplinary cooperation and to carry out scientific and standardized management of oral infectious diseases according to the particularity of the physiology of pregnant patients (Figure 1).

## Conclusion

There is still some controversy about the relationship between periodontal disease and adverse pregnancy outcomes, but numerous studies have shown a significant association between periodontal disease and preterm birth or low birth weight. Taken together, periodontal pathogen infection, the changes in the level of inflammatory mediators, host immune response, gene polymorphism, and APS have been recognized as common pathogenesis of periodontal disease and adverse pregnancy outcomes. Further exploration of the underlying mechanisms can provide more potential directions for their prevention and treatment. Appropriate supplementation of a variety of nutrition during pregnancy has become an effective way to prevent periodontal disease and adverse pregnancy outcomes and can assist in improving the effect of periodontal non-surgical treatment. Meanwhile, as an independent risk factor for adverse pregnancy outcomes, periodontal disease can be preventable and manageable. Preventive and restorative periodontal treatment during pregnancy is safe. It is recommended to finish routine oral examinations and deal with the oral infections before conceiving, so as to prevent women to suffer from periodontal disease during gestation. Meanwhlie, the management of periodontal infection during pregnancy should follow relevant treatment principles, and the ideal time for periodontal treatment is the second trimester (14–27 weeks). If intervention for infection is absolutely necessary, emergency treatment can be performed throughout pregnancy.

## Author contributions

JY: study design, manuscript research and preparation, and manuscript review. MN: original manuscript writing. LX: language modification. All authors contributed to the article and approved the submitted version.

## References

[1] Mealey BL Moritz AJ. Hormonal influences: effects of diabetes mellitus and endogenous female sex steroid hormones on the periodontium. *Periodontol 2000.* (2003) 32:59–81. 10.1046/j.0906-6713.2002.03206.x 12756034

[2] Xing W Xiping F Zhixin L. *The Fourth National Oral Health Epidemiological Survey Report[M].* Beijing: People’s Health Publishing House (2018).

[3] Figuero E Han YW Furuichi Y. Periodontal diseases and adverse pregnancy outcomes: mechanisms. *Periodontol 2000.* (2020) 83:175–88. 10.1111/prd.12295 32385886

[4] Terzic M Aimagambetova G Terzic S Radunovic M Bapayeva G Laganà AS. Periodontal pathogens and preterm birth: current knowledge and further interventions. *Pathogens.* (2021) 10:730. 10.3390/pathogens10060730 34207831 PMC8227634

[5] Manrique-Corredor EJ Orozco-Beltran D Lopez-Pineda A Quesada JA Gil-Guillen VF Carratala-Munuera C. Maternal periodontitis and preterm birth: systematic review and meta-analysis. *Community Dent Oral Epidemiol.* (2019) 47:243–51. 10.1111/cdoe.12450 30812054

[6] Blencowe H Krasevec J de Onis M Black RE An X Stevens GA National, regional, and worldwide estimates of low birthweight in 2015, with trends from 2000: a systematic analysis. *Lancet Glob Health.* (2019) 7:e849–60. 10.1016/s2214-109x(18)30565-5 31103470 PMC6560046

[7] Ma’ayeh M Rood KM Kniss D Costantine MM. Novel interventions for the prevention of preeclampsia. *Curr Hypertens Rep.* (2020) 22:17. 10.1007/s11906-020-1026-8 32052203 PMC8237364

[8] Khan KS Wojdyla D Say L Gülmezoglu AM Van Look PF. Who analysis of causes of maternal death: a systematic review. *Lancet.* (2006) 367:1066–74. 10.1016/s0140-6736(06)68397-9 16581405

[9] Offenbacher S Katz V Fertik G Collins J Boyd D Maynor G Periodontal infection as a possible risk factor for preterm low birth weight. *J Periodontol.* (1996) 67(Suppl 10S):1103–13. 10.1902/jop.1996.67.10s.1103 29539791

[10] Pockpa ZAD Soueidan A Koffi-Coulibaly NT Limam A Badran Z Struillou X. Periodontal diseases and adverse pregnancy outcomes: review of two decades of clinical research. *Oral Health Prev Dent.* (2021) 19:77–83. 10.3290/j.ohpd.b898969 33491381 PMC11641288

[11] Massaro CR Buratti M de Paula TNP Piana EA Wachter F Hoshi AT Maternal periodontal disease as a risk factor for preterm birth and low-birth-weight babies: a case-control study. *Gen Dent.* (2020) 68:44–9. 33136045

[12] Márquez-Corona ML Tellez-Girón-Valdez A Pontigo-Loyola AP Islas-Zarazúa R Robles-Bermeo NL Gonzalez-López BS Preterm birth associated with periodontal and dental indicators: a pilot case-control study in a developing country. *J Matern Fetal Neonatal Med.* (2021) 34:690–5. 10.1080/14767058.2019.1613363 31035800

[13] Lee YL Hu HY Chou SY Lin CL Cheng FS Yu CY Periodontal disease and preterm delivery: a nationwide population-based cohort study of Taiwan. *Sci Rep.* (2022) 12:3297. 10.1038/s41598-022-07425-8 35228672 PMC8885688

[14] Bhola S Geddis-Regan A. Could optimising periodontal health in expectant mothers reduce the risk of babies being born prematurely? *Evid Based Dent.* (2021) 22:14–5. 10.1038/s41432-021-0149-3 33772122

[15] de Oliveira LJC Cademartori MG Schuch HS Barros FC Silveira MF Correa MB Periodontal disease and preterm birth: findings from the 2015 pelotas birth cohort study. *Oral Dis.* (2021) 27:1519–27. 10.1111/odi.13670 33231907

[16] Uwambaye P Munyanshongore C Rulisa S Shiau H Nuhu A Kerr MS. Assessing the association between periodontitis and premature birth: a case-control study. *BMC Pregnancy Childbirth.* (2021) 21:204. 10.1186/s12884-021-03700-0 33711951 PMC7953642

[17] Micu IC Roman A Ticala F Soanca A Ciurea A Objelean A Relationship between preterm birth and post-partum periodontal maternal status: a hospital-based romanian study. *Arch Gynecol Obstet.* (2020) 301:1189–98. 10.1007/s00404-020-05521-6 32274638

[18] Mahapatra A Nayak R Satpathy A Pati BK Mohanty R Mohanty G Maternal periodontal status, oral inflammatory load, and systemic inflammation are associated with low infant birth weight. *J Periodontol.* (2021) 92:1107–16. 10.1002/jper.20-0266 33155287

[19] Tanneeru S Mahendra J Shaik MV. Evaluation of microflora (viral and bacterial) in subgingival and placental samples of pregnant women with preeclampsia with and without periodontal disease: a cross-sectional study. *J Int Soc Prev Community Dent.* (2020) 10:171–6. 10.4103/jispcd.JISPCD_341_19 32670905 PMC7339998

[20] Mata K Nobre AVV Felix Silva PH Oliezer RS Fernandes C Amaral J A new mixed model of periodontitis-induced preeclampsia: a pilot study. *J Periodontal Res.* (2021) 56:726–34. 10.1111/jre.12869 33686671

[21] Calixto NR Alves CM Abreu LM Thomaz EB Vidal FC Filho IS Detection of periodontal pathogens in mothers of preterm birth and/or low weight. *Med Oral Patol Oral Cir Bucal.* (2019) 24:e776–81. 10.4317/medoral.23135 31655839 PMC6901144

[22] Caneiro L Lopez-Carral JM Martin-Lancharro P Linares A Batalla P Blanco-Carrion J. Periodontitis as a preterm birth risk factor in caucasian women: a cohort study. *Oral Health Prev Dent.* (2020) 18:77–84. 10.3290/j.ohpd.a44116 32051974 PMC11654515

[23] Ye C Kapila Y. Oral microbiome shifts during pregnancy and adverse pregnancy outcomes: hormonal and immunologic changes at play. *Periodontol 2000.* (2021) 87:276–81. 10.1111/prd.12386 34463984 PMC8457099

[24] Jang H Patoine A Wu TT Castillo DA Xiao J. Oral microflora and pregnancy: a systematic review and meta-analysis. *Sci Rep.* (2021) 11:16870. 10.1038/s41598-021-96495-1 34413437 PMC8377136

[25] Mei F Xie M Huang X Long Y Lu X Wang X Porphyromonas gingivalis and its systemic impact: current status. *Pathogens.* (2020) 9:944. 10.3390/pathogens9110944 33202751 PMC7696708

[26] Vanterpool SF Been JV Houben ML Nikkels PG De Krijger RR Zimmermann LJ Porphyromonas gingivalis within placental villous mesenchyme and umbilical cord stroma is associated with adverse pregnancy outcome. *PLoS One.* (2016) 11:e0146157. 10.1371/journal.pone.0146157 26731111 PMC4701427

[27] Inaba H Amano A Lamont RJ Murakami Y Matsumoto-Nakano M. Cell cycle arrest and apoptosis induced by porphyromonas gingivalis require jun N-terminal protein kinase- and P53-mediated P38 activation in human trophoblasts. *Infect Immun.* (2018) 86:e00923-17. 10.1128/iai.00923-17 29339463 PMC5865039

[28] Guo H Ren H Liang S Ji Y Jiang H Zhang P Phosphatidylinositol 3-kinase/Akt signal pathway resists the apoptosis and inflammation in human extravillous trophoblasts induced by porphyromonas gingivalis. *Mol Immunol.* (2018) 104:100–7. 10.1016/j.molimm.2018.10.008 30448607

[29] Chopra A Radhakrishnan R Sharma M. Porphyromonas gingivalis and adverse pregnancy outcomes: a review on its intricate pathogenic mechanisms. *Crit Rev Microbiol.* (2020) 46:213–36. 10.1080/1040841x.2020.1747392 32267781

[30] Gómez LA De Avila J Castillo DM Montenegro DA Trujillo TG Suárez LJ Porphyromonas gingivalis placental atopobiosis and inflammatory responses in women with adverse pregnancy outcomes. *Front Microbiol.* (2020) 11:591626. 10.3389/fmicb.2020.591626 33343532 PMC7738622

[31] Whitley GS Cartwright JE. Trophoblast-mediated spiral artery remodelling: a role for apoptosis. *J Anat.* (2009) 215:21–6. 10.1111/j.1469-7580.2008.01039.x 19215319 PMC2714635

[32] Saadaoui M Singh P Al Khodor S. Oral microbiome and pregnancy: a bidirectional relationship. *J Reprod Immunol.* (2021) 145:103293. 10.1016/j.jri.2021.103293 33676065

[33] Fardini Y Wang X Témoin S Nithianantham S Lee D Shoham M *Fusobacterium Nucleatum* adhesin fada binds vascular endothelial cadherin and alters endothelial integrity. *Mol Microbiol.* (2011) 82:1468–80. 10.1111/j.1365-2958.2011.07905.x 22040113 PMC3237733

[34] Wu C Chen YW Scheible M Chang C Wittchen M Lee JH Genetic and molecular determinants of polymicrobial interactions in *Fusobacterium Nucleatum*. *Proc Natl Acad Sci USA.* (2021) 118:e2006482118. 10.1073/pnas.2006482118 34074747 PMC8201914

[35] Coppenhagen-Glazer S Sol A Abed J Naor R Zhang X Han YW Fap2 of *Fusobacterium Nucleatum* is a galactose-inhibitable adhesin involved in coaggregation, cell adhesion, and preterm birth. *Infect Immun.* (2015) 83:1104–13. 10.1128/iai.02838-14 25561710 PMC4333458

[36] Vander Haar EL So J Gyamfi-Bannerman C Han YW. *Fusobacterium Nucleatum* and adverse pregnancy outcomes: epidemiological and mechanistic evidence. *Anaerobe.* (2018) 50:55–9. 10.1016/j.anaerobe.2018.01.008 29409815 PMC6750227

[37] Latorre Uriza C Velosa-Porras J Roa NS Quiñones Lara SM Silva J Ruiz AJ Periodontal disease, inflammatory cytokines, and Pge(2) in pregnant patients at risk of preterm delivery: a pilot study. *Infect Dis Obstet Gynecol.* (2018) 2018:7027683. 10.1155/2018/7027683 30154640 PMC6093048

[38] Sanz M Kornman K. Periodontitis and adverse pregnancy outcomes: consensus report of the joint Efp/Aap workshop on periodontitis and systemic diseases. *J Periodontol.* (2013) 84(4 Suppl):S164–9. 10.1902/jop.2013.1340016 23631576

[39] Penova-Veselinovic B Keelan JA Wang CA Newnham JP Pennell CE. Changes in inflammatory mediators in gingival crevicular fluid following periodontal disease treatment in pregnancy: relationship to adverse pregnancy outcome. *J Reprod Immunol.* (2015) 112:1–10. 10.1016/j.jri.2015.05.002 26093363

[40] Perunovic N Rakic MM Nikolic LI Jankovic SM Aleksic ZM Plecas DV The association between periodontal inflammation and labor triggers (elevated cytokine levels) in preterm birth: a cross-sectional study. *J Periodontol.* (2016) 87:248–56. 10.1902/jop.2015.150364 26447753

[41] Chaparro A Sanz A Quintero A Inostroza C Ramirez V Carrion F Increased inflammatory biomarkers in early pregnancy is associated with the development of pre-eclampsia in patients with periodontitis: a case control study. *J Periodontal Res.* (2013) 48:302–7. 10.1111/jre.12008 23035752

[42] Liang S Ren H Guo H Xing W Liu C Ji Y Periodontal infection with porphyromonas gingivalis induces preterm birth and lower birth weight in rats. *Mol Oral Microbiol.* (2018) 33:312–21. 10.1111/omi.12227 29754448

[43] Konishi H Urabe S Teraoka Y Morishita Y Koh I Sugimoto J Porphyromonas gingivalis, a cause of preterm birth in mice, induces an inflammatory response in human amnion mesenchymal cells but not epithelial cells. *Placenta.* (2020) 99:21–6. 10.1016/j.placenta.2020.07.016 32738645

[44] Madianos PN Bobetsis YA Offenbacher S. Adverse pregnancy outcomes (Apos) and periodontal disease: pathogenic mechanisms. *J Periodontol.* (2013) 84(4 Suppl):S170–80. 10.1902/jop.2013.1340015 23631577

[45] Reddy BV Tanneeru S Chava VK. The effect of phase-i periodontal therapy on pregnancy outcome in chronic periodontitis patients. *J Obstet Gynaecol.* (2014) 34:29–32. 10.3109/01443615.2013.829029 24359045

[46] Ye C Xia Z Tang J Khemwong T Kapila Y Kuraji R Unculturable and culturable periodontal-related bacteria are associated with periodontal inflammation during pregnancy and with preterm low birth weight delivery. *Sci Rep.* (2020) 10:15807. 10.1038/s41598-020-72807-9 32978483 PMC7519089

[47] Ebersole JL Novak MJ Michalowicz BS Hodges JS Steffen MJ Ferguson JE Systemic immune responses in pregnancy and periodontitis: relationship to pregnancy outcomes in the obstetrics and periodontal therapy (Opt) study. *J Periodontol.* (2009) 80:953–60. 10.1902/jop.2009.080464 19485826 PMC4133130

[48] Lin D Moss K Beck JD Hefti A Offenbacher S. Persistently high levels of periodontal pathogens associated with preterm pregnancy outcome. *J Periodontol.* (2007) 78:833–41. 10.1902/jop.2007.060201 17470016

[49] Ye C Katagiri S Miyasaka N Kobayashi H Khemwong T Nagasawa T The periodontopathic bacteria in placenta, saliva and subgingival plaque of threatened preterm labor and preterm low birth weight cases: a longitudinal study in japanese pregnant women. *Clin Oral Investig.* (2020) 24:4261–70. 10.1007/s00784-020-03287-4 32333174

[50] Dasanayake AP Boyd D Madianos PN Offenbacher S Hills E. The association between porphyromonas gingivalis-specific maternal serum igg and low birth weight. *J Periodontol.* (2001) 72:1491–7. 10.1902/jop.2001.72.11.1491 11759860

[51] Sugita N Iwanaga R Kobayashi T Yoshie H. Association of the Fcγriib-Nt645+25a/G polymorphism with the expression level of the fcγriib receptor, the antibody response to porphyromonas gingivalis and the severity of periodontitis. *J Periodontal Res.* (2012) 47:105–13. 10.1111/j.1600-0765.2011.01411.x 21906057

[52] Wang Y Sugita N Kikuchi A Iwanaga R Hirano E Shimada Y Fcγriib-Nt645+25a/G gene polymorphism and periodontitis in japanese women with preeclampsia. *Int J Immunogenet.* (2012) 39:492–500. 10.1111/j.1744-313X.2012.01124.x 22594540

[53] Schenkein HA Thomas RR. Anticardiolipin (Acl) in sera from periodontitis subjects activate toll-like receptor 4 (Tlr4). *PLoS One.* (2018) 13:e0203494. 10.1371/journal.pone.0203494 30192824 PMC6128564

[54] Xourgia E Tektonidou MG. An update on antiphospholipid syndrome. *Curr Rheumatol Rep.* (2022) 23:84. 10.1007/s11926-021-01051-5 34985625

[55] Ye C Katagiri S Miyasaka N Bharti P Kobayashi H Takeuchi Y The anti-phospholipid antibody-dependent and independent effects of periodontopathic bacteria on threatened preterm labor and preterm birth. *Arch Gynecol Obstet.* (2013) 288:65–72. 10.1007/s00404-013-2741-z 23400354

[56] Blank M Krause I Fridkin M Keller N Kopolovic J Goldberg I Bacterial induction of autoantibodies to beta2-glycoprotein-i accounts for the infectious etiology of antiphospholipid syndrome. *J Clin Invest.* (2002) 109:797–804. 10.1172/jci12337 11901188 PMC150905

[57] Schenkein HA Thomas RR. Anticardiolipin from periodontitis patients impact fetal loss and annexin V. *J Dent Res.* (2020) 99:797–803. 10.1177/0022034520913244 32202953 PMC7313349

[58] Xie H Sheng L Zhou H Yan J. The role of Tlr4 in pathophysiology of antiphospholipid syndrome-associated thrombosis and pregnancy morbidity. *Br J Haematol.* (2014) 164:165–76. 10.1111/bjh.12587 24180619

[59] Colasanti T Alessandri C Capozzi A Sorice M Delunardo F Longo A Autoantibodies specific to a peptide of B 2-glycoprotein I cross-react with Tlr4, inducing a proinflammatory phenotype in endothelial cells and monocytes. *Blood.* (2012) 120:3360–70. 10.1182/blood-2011-09-378851 22932793

[60] Singleton CR Li Y Odoms-Young A Zenk SN Powell LM. Change in food and beverage availability and marketing following the introduction of a healthy food financing initiative-supported supermarket. *Am J Health Promot.* (2019) 33:525–33. 10.1177/0890117118801744 30282461

[61] Martinon P Fraticelli L Giboreau A Dussart C Bourgeois D Carrouel F. Nutrition as a key modifiable factor for periodontitis and main chronic diseases. *J Clin Med.* (2021) 10:197. 10.3390/jcm10020197 33430519 PMC7827391

[62] Lee HJ Ha JE Bae KH. Synergistic effect of maternal obesity and periodontitis on preterm birth in women with pre-eclampsia: a prospective study. *J Clin Periodontol.* (2016) 43:646–51. 10.1111/jcpe.12574 27167920

[63] Liu J Wu Y Wang B Yuan X Fang B. High levels of glucose induced the caspase-3/parp signaling pathway, leading to apoptosis in human periodontal ligament fibroblasts. *Cell Biochem Biophys.* (2013) 66:229–37. 10.1007/s12013-012-9470-y 23161104

[64] Lu MS He JR Chen Q Lu J Wei X Zhou Q Maternal dietary patterns during pregnancy and preterm delivery: a large prospective cohort study in China. *Nutr J.* (2018) 17:71. 10.1186/s12937-018-0377-3 30045719 PMC6060524

[65] Sanjarimoghaddam F Bahadori F Bakhshimoghaddam F Alizadeh M. Association between quality and quantity of dietary carbohydrate and pregnancy-induced hypertension: a case-control study. *Clin Nutr ESPEN.* (2019) 33:158–63. 10.1016/j.clnesp.2019.06.001 31451254

[66] Azuma MM Cardoso CBM da Silva CC de Oliveira PHC Jacinto RC Andrada AC The use of omega-3 fatty acids in the treatment of oral diseases. *Oral Dis.* (2022) 28:264–74. 10.1111/odi.13667 33022782

[67] Chatterjee D Chatterjee A Kalra D Kapoor A Vijay S Jain S. Role of adjunct use of omega 3 fatty acids in periodontal therapy of periodontitis. a systematic review and meta-analysis. *J Oral Biol Craniofac Res.* (2022) 12:55–62. 10.1016/j.jobcr.2021.10.005 34760614 PMC8566999

[68] Ozaki Y Morozumi T Watanabe K Toyama T Sasaki H Sato T Inhibitory effect of omega-3 fatty acids on alveolar bone resorption and osteoclast differentiation. *J Oral Sci.* (2020) 62:298–302. 10.2334/josnusd.19-0267 32581177

[69] Simmonds LA Sullivan TR Skubisz M Middleton PF Best KP Yelland LN Omega-3 fatty acid supplementation in pregnancy-baseline omega-3 status and early preterm birth: exploratory analysis of a randomised controlled trial. *Bjog.* (2020) 127:975–81. 10.1111/1471-0528.16168 32034969

[70] Bakouei F Delavar MA Mashayekh-Amiri S Esmailzadeh S Taheri Z. Efficacy of N-3 fatty acids supplementation on the prevention of pregnancy induced-hypertension or preeclampsia: a systematic review and meta-analysis. *Taiwan J Obstet Gynecol.* (2020) 59:8–15. 10.1016/j.tjog.2019.11.002 32039806

[71] Garcia-So J Zhang X Yang X Rubinstein MR Mao Y Kitajewski J Omega-3 fatty acids suppress *Fusobacterium Nucleatum*-induced placental inflammation originating from maternal endothelial cells. *JCI Insight.* (2019) 4:e125436. 10.1172/jci.insight.125436 30728337 PMC6413831

[72] Dodington DW Fritz PC Sullivan PJ Ward WE. Higher intakes of fruits and vegetables, B -carotene, vitamin C, A -tocopherol, Epa, and Dha are positively associated with periodontal healing after nonsurgical periodontal therapy in nonsmokers but not in smokers. *J Nutr.* (2015) 145:2512–9. 10.3945/jn.115.211524 26423734

[73] Dommisch H Kuzmanova D Jönsson D Grant M Chapple I. Effect of micronutrient malnutrition on periodontal disease and periodontal therapy. *Periodontol 2000.* (2018) 78:129–53. 10.1111/prd.12233 30198127

[74] McCauley ME van den Broek N Dou L Othman M. Vitamin a supplementation during pregnancy for maternal and newborn outcomes. *Cochrane Database Syst Rev.* (2015) 2015:Cd008666. 10.1002/14651858.CD008666.pub3 26503498 PMC7173731

[75] Duan S Jiang Y Mou K Wang Y Zhou S Sun B. Correlation of serum vitamin a and vitamin e levels with the occurrence and severity of preeclampsia. *Am J Transl Res.* (2021) 13:14203–10. 35035766 PMC8748077

[76] Zong G Holtfreter B Scott AE Völzke H Petersmann A Dietrich T Serum vitamin B12 is inversely associated with periodontal progression and risk of tooth loss: a prospective cohort study. *J Clin Periodontol.* (2016) 43:2–9. 10.1111/jcpe.12483 26613385

[77] Bulloch RE Lovell AL Jordan VMB McCowan LME Thompson JMD Wall CR. Maternal folic acid supplementation for the prevention of preeclampsia: a systematic review and meta-analysis. *Paediatr Perinat Epidemiol.* (2018) 32:346–57. 10.1111/ppe.12476 29882975

[78] Rogne T Tielemans MJ Chong MF Yajnik CS Krishnaveni GV Poston L Associations of maternal vitamin B12 concentration in pregnancy with the risks of preterm birth and low birth weight: a systematic review and meta-analysis of individual participant data. *Am J Epidemiol.* (2017) 185:212–23. 10.1093/aje/kww212 28108470 PMC5390862

[79] Varela-López A Navarro-Hortal MD Giampieri F Bullón P Battino M Quiles JL. Nutraceuticals in periodontal health: a systematic review on the role of vitamins in periodontal health maintenance. *Molecules.* (2018) 23:1226. 10.3390/molecules23051226 29783781 PMC6099579

[80] Mousa A Naqash A Lim S. Macronutrient and micronutrient intake during pregnancy: an overview of recent evidence. *Nutrients.* (2019) 11:443. 10.3390/nu11020443 30791647 PMC6413112

[81] Tada A Miura H. The relationship between vitamin c and periodontal diseases: a systematic review. *Int J Environ Res Public Health.* (2019) 16:2472. 10.3390/ijerph16142472 31336735 PMC6678404

[82] Shetty MS Ramesh A Shetty PK Agumbe P. Salivary and serum antioxidants in women with preeclampsia with or without periodontal disease. *J Obstet Gynaecol India.* (2018) 68:33–8. 10.1007/s13224-017-0993-4 29391673 PMC5783910

[83] Agrawal AA Kolte AP Kolte RA Chari S Gupta M Pakhmode R. Evaluation and comparison of serum vitamin d and calcium levels in periodontally healthy, chronic gingivitis and chronic periodontitis in patients with and without diabetes mellitus - a cross-sectional study. *Acta Odontol Scand.* (2019) 77:592–9. 10.1080/00016357.2019.1623910 31198072

[84] Rodrigues Amorim Adegboye A Dias Santana D Cocate PG Benaim C Teixeira Dos Santos PP Heitmann BL Vitamin D and calcium milk fortification in pregnant women with periodontitis: a feasibility trial. *Int J Environ Res Public Health.* (2020) 17:8023. 10.3390/ijerph17218023 33143369 PMC7662458

[85] Rodrigues Amorim Adegboye A Dias Santana D Teixeira Dos Santos PP Guedes Cocate P Benaim C Trindade de Castro MB Exploratory efficacy of calcium-vitamin d milk fortification and periodontal therapy on maternal oral health and metabolic and inflammatory profile. *Nutrients.* (2021) 13:783. 10.3390/nu13030783 33673568 PMC7997467

[86] Ferrillo M Migliario M Roccuzzo A Molinero-Mourelle P Falcicchio G Umano GR Periodontal disease and vitamin D deficiency in pregnant women: which correlation with preterm and low-weight birth? *J Clin Med.* (2021) 10:4578. 10.3390/jcm10194578 34640596 PMC8509337

[87] Taneja A Gupta S Kaur G Jain NP Kaur J Kaur S. Vitamin D: its deficiency and effect of supplementation on maternal outcome. *J Assoc Physicians India.* (2020) 68:47–50. 32138484

[88] Black RE Allen LH Bhutta ZA Caulfield LE de Onis M Ezzati M Maternal and child undernutrition: global and regional exposures and health consequences. *Lancet.* (2008) 371:243–60. 10.1016/s0140-6736(07)61690-0 18207566

[89] Marshall NE Abrams B Barbour LA Catalano P Christian P Friedman JE The importance of nutrition in pregnancy and lactation: lifelong consequences. *Am J Obstet Gynecol.* (2022) 226:607–32. 10.1016/j.ajog.2021.12.035 34968458 PMC9182711

[90] Nguyen T Brody H Radaic A Kapila Y. Probiotics for periodontal health-current molecular findings. *Periodontol 2000.* (2021) 87:254–67. 10.1111/prd.12382 34463979 PMC8448672

[91] Bustamante M Oomah BD Mosi-Roa Y Rubilar M Burgos-Díaz C. Probiotics as an adjunct therapy for the treatment of halitosis, dental caries and periodontitis. *Probiotics Antimicrob Proteins.* (2020) 12:325–34. 10.1007/s12602-019-9521-4 30729452

[92] López-Moreno A Aguilera M. Probiotics dietary supplementation for modulating endocrine and fertility microbiota dysbiosis. *Nutrients.* (2020) 12:757. 10.3390/nu12030757 32182980 PMC7146451

[93] López-Moreno A Aguilera M. Vaginal probiotics for reproductive health and related dysbiosis: systematic review and meta-analysis. *J Clin Med.* (2021) 10:1461. 10.3390/jcm10071461 33918150 PMC8037567

[94] Bi WG Emami E Luo ZC Santamaria C Wei SQ. Effect of periodontal treatment in pregnancy on perinatal outcomes: a systematic review and meta-analysis. *J Matern Fetal Neonatal Med.* (2021) 34 :3259–68. 10.1080/14767058.2019.1678142 31630597

[95] Govindasamy R Periyasamy S Narayanan M Balaji VR Dhanasekaran M Karthikeyan B. The influence of nonsurgical periodontal therapy on the occurrence of adverse pregnancy outcomes: a systematic review of the current evidence. *J Indian Soc Periodontol.* (2020) 24:7–14. 10.4103/jisp.jisp_228_19 31983838 PMC6961443

[96] Dave BH Shah EB Gaikwad RV Shah SS. Association of preterm low-birth-weight infants and maternal periodontitis during pregnancy: an interventional study. *J Indian Soc Pedod Prev Dent.* (2021) 39:183–8. 34341239 10.4103/jisppd.jisppd_270_20

[97] Caneiro-Queija L López-Carral J Martin-Lancharro P Limeres-Posse J Diz-Dios P Blanco-Carrion J. Non-surgical treatment of periodontal disease in a pregnant caucasian women population: adverse pregnancy outcomes of a randomized clinical trial. *Int J Environ Res Public Health.* (2019) 16:3638. 10.3390/ijerph16193638 31569780 PMC6801449

[98] López NJ Uribe S Martinez B. Effect of periodontal treatment on preterm birth rate: a systematic review of meta-analyses. *Periodontol 2000.* (2015) 67:87–130. 10.1111/prd.12073 25494599

[99] Xu B Han YW. Oral Bacteria, oral health, and adverse pregnancy outcomes. *Periodontol 2000.* (2022) 89:181–9. 10.1111/prd.12436 35244963

[100] Favero V Bacci C Volpato A Bandiera M Favero L Zanette G. Pregnancy and dentistry: a literature review on risk management during dental surgical procedures. *Dent J.* (2021) 9:46. 10.3390/dj9040046 33921608 PMC8072957

